# Metal–organic framework composites with luminescent pincer platinum(ii) complexes: ^3^MMLCT emission and photoinduced dehydrogenation catalysis†
†Electronic supplementary information (ESI) available: Experimental details. See DOI: 10.1039/c7sc04528j


**DOI:** 10.1039/c7sc04528j

**Published:** 2018-02-01

**Authors:** Chun-Yi Sun, Wai-Pong To, Faan-Fung Hung, Xin-Long Wang, Zhong-Min Su, Chi-Ming Che

**Affiliations:** a State Key Laboratory of Synthetic Chemistry , Institute of Molecular Functional Materials , HKU-CAS Joint Laboratory on New Materials , Department of Chemistry , The University of Hong Kong , Pokfulam Road , Hong Kong , China . Email: cmche@hku.hk; b HKU Shenzhen Institute of Research and Innovation , Shenzhen , Guangdong 518053 , China; c Department of Chemistry , Northeast Normal University , Changchun , Jilin , 130024 China

## Abstract

Pincer platinum(ii) complexes are well documented to exhibit weak intermolecular interactions in the solid state and 77 K glassy solutions, leading to emissive triplet metal–metal-to-ligand charge transfer (^3^MMLCT) excited states that often vanish in dilute solutions. In this work, metal–organic framework (MOF) materials are introduced to provide a “solid solution” environment for easy access to ^3^MMLCT excited states of pincer platinum(ii) complexes. Phosphorescent composites Pt^II^@MOFs (**1–4**) with matrix-dependent monomers and oligomer emission properties were obtained. These Pt^II^@MOFs are efficient catalysts for photoinduced dehydrogenation reactions.

## Abstract 2

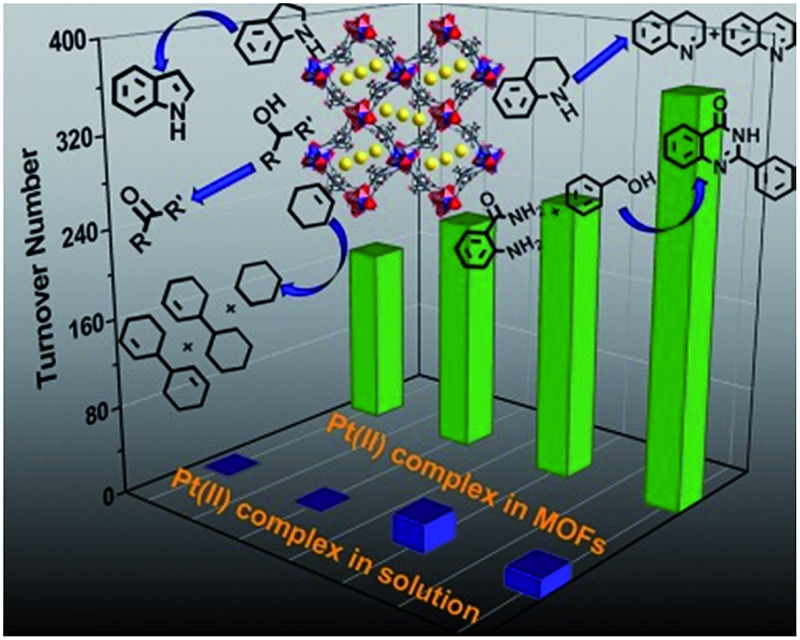
Metal–organic framework materials are introduced to provide a “solid solution” environment for easy access to metal–metal-to-ligand charge transfer excited states of pincer platinum(ii) complexes and act as photocatalysts.

## Introduction

Platinum(ii) complexes are known to exhibit weak intramolecular and intermolecular interactions that lead to triplet metal–metal-to-ligand charge transfer (^3^MMLCT) and/or ^3^[5dσ* → 6pσ] excited states.^1^,^2^ The diradical character of such excited states with enhanced metal–metal bonding interactions renders these complexes capable of performing photocatalytic C–X bond cleaving reactions.^3^,^4^ The classic example, [Pt_2_(μ-P_2_O_5_H_2_)_4_]^4–^, is a highly active catalyst for the photoinduced dehydrogenation of alcohols to aldehydes/ketones in the absence of a sacrificial electron acceptor *via* its long-lived ^3^[5dσ*6pσ] excited state.1*a* Due to the uniqueness of the μ-pyrophosphito ligand, extending the photochemistry of [Pt_2_(μ-P_2_O_5_H_2_)_4_]^4–^ to other platinum(ii) complexes is a non-trivial task. In this regard, pincer Pt^II^ complexes are appealing alternatives because their structures can be readily modified to elicit intermolecular Pt···Pt interactions and hence emissive ^3^MMLCT excited states in concentrated solutions or in the solid state.^5^ However, the photophysical and photochemical properties associated with the ^3^MMLCT excited states of Pt^II^ complexes often vanish in dilute solutions.

Metal–organic frameworks (MOFs) have emerged as a new class of highly promising porous materials.^6^,^7^ In particular, the porous environment in MOFs provides a unique platform to confine and stabilize guest species, and as a result, novel properties of the incorporated guest may emerge.^8^ In the literature, reports on the incorporation of Pt^II^ complexes into MOFs are sparse;^9^ these previously reported Pt^II^-MOF composites were formed by the coordination of Pt^II^Cl_2_ (ref. 9*a–d*) or Pt^II^L_2_ (L_2_ = 2,2′-bipyridine, (OPPh_3_)_2_ or (PPh_3_)_2_)9*e* moieties to the bipyridine units of MOFs and in some cases they were studied as photocatalysts for hydrogen production from water.9a,c,d We envisage that incorporating luminescent pincer Pt^II^ complexes into the pores of MOFs by, for example, a cation exchange method, can be a strategy to develop the ^3^MMLCT photochemistry of platinum(ii) complexes. This method has been shown to be effective in placing phosphorescent d^6^ and d^8^ metal complexes inside MOFs.^10^,^11^ In this work, Pt^II^ complexes with a pincer C⁁N⁁C (where C is an N-heterocyclic carbene) ligand, [Pt(C⁁N⁁C)(C≡CC_6_H_5_)]^+^ (**Pt1**) and [Pt(C⁁N⁁C)(C≡N)]^+^ (**Pt2**; both **Pt1** and **Pt2** have PF_6_^–^ as a counteranion) were synthesized and used as guest species for three MOFs with different porous structures. These Pt^II^@MOF composites were found to display matrix-dependent emission properties with emission peak maxima (*λ*_max_) ranging from 450 to 625 nm in air and also to catalyze photoinduced dehydrogenation reactions of various organic compounds with activities higher than those of the corresponding Pt^II^ complexes in homogeneous solutions by an order of magnitude.

## Results and discussion

### Syntheses and characterization of Pt^II^@MOFs

**Fig. 1:**
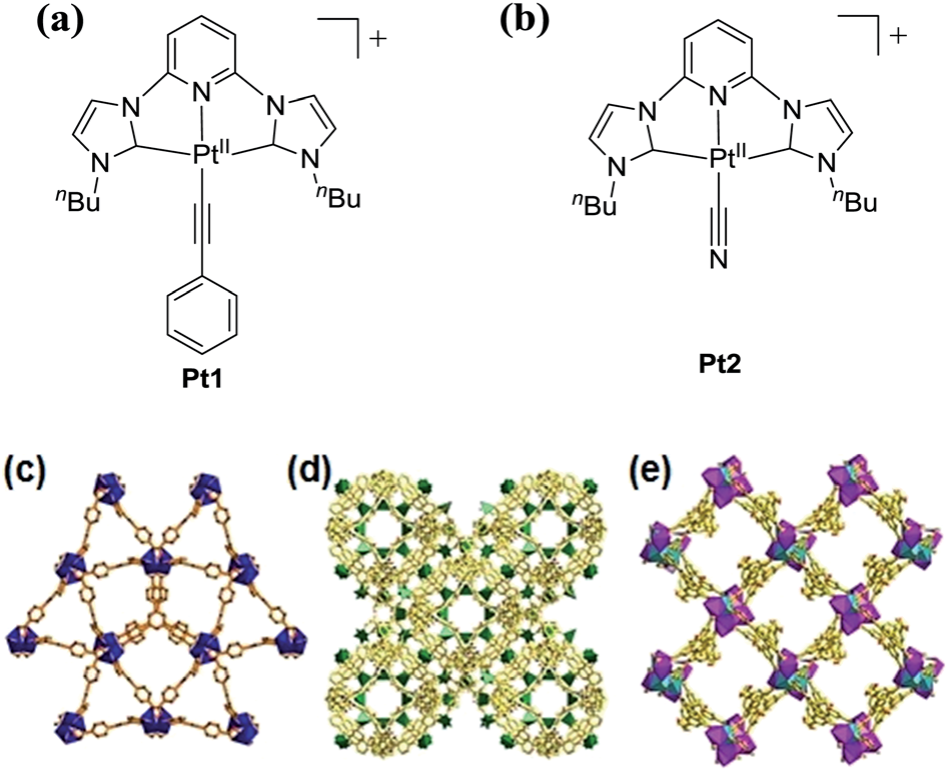
**Pt1** (a) and **Pt2** (b) complexes used in this work. 3D polyhedral structures of ZJU-28 (c), MOF1 (d) and MOF2 (e), with C shown in gray, O red, and N blue.

**Pt1** and **Pt2** (Fig. 1a and b),^12^ and host MOFs ZJU-28,^13^ MOF1 14 and MOF2 15 featuring negatively charged frameworks,^13^–^15^ were synthesized according to previously reported procedures. The molecular sizes of **Pt1** and **Pt2** based on optimization from theoretical calculations are 12.15 × 8.05 and 8.05 × 7.95 Å^2^, respectively. The formulas of MOF1, MOF2, and ZJU-28 are [(CH_3_)_2_NH_2_]_2_[Zn(TATAT)_2/3_]·3DMF·H_2_O, [(CH_3_)_2_NH_2_]_2_[ZnNa_2_(μ_2_-H_2_O)_2_(H_2_O)_2_(TATAT)]·2DMF, and [(CH_3_)_2_NH_2_]_3_[In_3_(BTB)_4_]·12DMF·22H_2_O, respectively (TATAT = 5,5′,5′′-(1,3,5-triazine-2,4,6-triyl)tris(azanediyl)triisophthalate; BTB = 4,4′,4′′-benzene-1,3,5-triyl-tribenzoate). Single-crystal X-ray structure determination revealed that ZJU-28 is a framework of parallel interwoven corrugated 6^3^ nets containing two types of 1D channel (Fig. 1c) with a maximal pore size of 14.7 × 9.8 Å^2^.^13^ MOF1 shows a 3D chiral framework featuring an alternating arrangement of hexagonal and trigonal prismatic cages (Fig. 1d) with a maximal window size of 14.3 × 11.5 Å^2^,^14^ and MOF2 has a chiral framework with metal–organic nanotubes formed by heterometallic helical rods (Fig. 1e) and a channel size of 17.0 × 23.0 Å^2^.^15^

Pt^II^@MOFs with different loadings of Pt^II^ complexes, namely, Pt1@ZJU-28 (**1a–1e**), Pt1@MOF1 (**2a–2e**), Pt1@MOF2 (**3a–3e**) and Pt2@ZJU-28 (**4a–4e**), were obtained as yellow or pale yellow solids (Fig. S1, ESI†) by immersing MOF crystals in DMF or MeCN solutions of the Pt^II^ complexes at different concentrations ((0.25–10) × 10^–4^ M). **Pt2** was found to be unstable upon incorporation into MOF1 or MOF2, which precluded studies on the Pt2@MOF1 and Pt2@MOF2 composites. The powder X-ray diffraction (PXRD) data of these composites were nearly identical to those of their matrix MOFs, indicating that the ion exchange process does not affect the crystallinity of the host materials (Fig. S2, ESI†). Inductively coupled plasma (ICP) mass spectrometric (MS) measurements (Table 1) showed that the loadings of the Pt^II^ complexes ranged from 0.08 to 8.68 wt%. Distribution of the Pt^II^ complex cation in a MOF was examined by optical microscopy, scanning electron microscope (SEM) imaging, energy dispersive X-ray (EDX) elemental mapping and N_2_ sorption experiments. Analysis of a cross section of a crystal of **2e** under an optical microscope showed that all of the surfaces of the split crystal emitted yellow light under light irradiation at 365 nm, indicating uniform distribution of **Pt1** throughout the crystal (Fig. S3, ESI†). SEM imaging and EDX elemental mapping in a cross section of a split crystal of Pt^II^@MOFs showed the Pt element to have random distribution in the inner space of the Pt^II^@MOFs (Fig. S4–S7, ESI†). Simulation by DFT calculation for the Pt^II^@MOFs, taking Pt1@MOF1 (**2**) as an example, revealed that the Pt^II^ complex resides close to the ligand of MOF and the distance between the pyridine ring in the C⁁N⁁C ligand of the Pt^II^ complex and the ligand of the MOF is ∼3.3 Å (Fig. S8, ESI†), with the adsorption energy being 8.270 eV. N_2_ sorption measurements revealed that the Brunauer–Emmett–Teller (BET) surface area decreased by >26% on going from MOF1 to **2e** (1112 → 818 m^2^ g^–1^, Fig. S9, ESI†), supporting the confinement of **Pt1** in the pores/channels of MOF1.

**Table 1:** Loadings of Pt^II^ complexes in Pt^II^@MOFs determined by ICP-MS

Pt^II^@MOFs	Loading of Pt^II^ complexes (wt%)				
Pt1@ZJU-28	**1a**	**1b**	**1c**	**1d**	**1e**
	0.11	0.28	0.54	1.36	1.50
Pt1@MOF1	**2a**	**2b**	**2c**	**2d**	**2e**
	0.08	0.35	0.64	1.82	3.45
Pt1@MOF2	**3a**	**3b**	**3c**	**3d**	**3e**
	0.60	1.37	2.82	5.92	8.68
Pt2@ZJU-28	**4a**	**4b**	**4c**	**4d**	**4e**
	0.19	0.27	0.81	1.32	2.41

### Spectroscopy and photophysical measurements

**Fig. 2:**
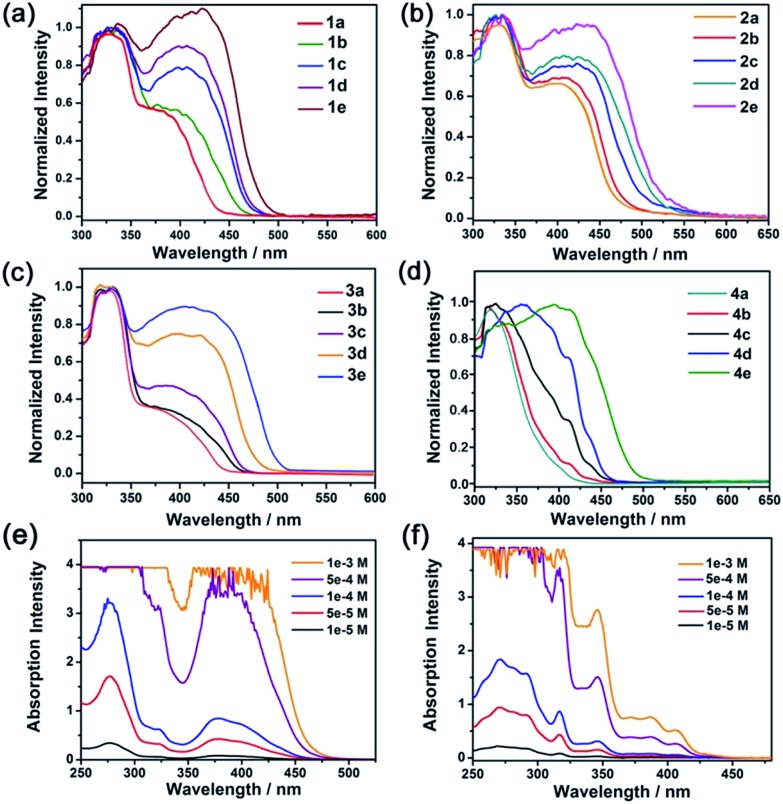
Electronic absorption spectra of Pt^II^@MOFs (a–d) and electronic absorption spectra of **Pt1** (e) and **Pt2** (f) in MeCN.

The electronic absorption spectra of composites **1**, **2** and **3** (Fig. 2, Table 2) with low complex concentrations showed intense absorption bands at 325, 327 and 330 nm, and moderately intense bands at 390, 405 and 400 nm, respectively. The high energy absorption bands are assigned to the absorption of the matrix MOFs and intraligand (^1^IL) π → π* transitions of the –C≡CC_6_H_5_ and C⁁N⁁C pincer ligands, whereas the lower-energy bands are assigned to the mixed singlet metal-to-ligand charge transfer (^1^MLCT) [dπ(Pt) → π*(C⁁N⁁C)] and the alkynyl-to-C⁁N⁁C ligand-to-ligand charge transfer (LLCT) [π(–C≡CC_6_H_5_) → π*(C⁁N⁁C)] transitions, both of which are characteristic absorptions of monomeric pincer Pt^II^ complexes in solution.^12^ Notably, a redshift of the low energy absorption band was observed in composites with a higher loading of the Pt^II^ complex. For example, composite **4a** ([Pt^II^] = 0.19%; Fig. 2d) showed absorption at only 300–400 nm. For composites **4d** and **4e** with higher [Pt^II^] loadings of 1.32 and 2.41 wt%, respectively, there was a new, broad absorption band at 400–500 nm attributable to a ^1^MMLCT transition of aggregated species of **Pt2** (ref. 12) inside the MOF (Fig. 2d). For comparison, increasing the concentration of **Pt2** in solution from 5 × 10^–5^ M to 1 × 10^–3^ M did not result in a notable shift in the absorption peak maxima or the formation of a new absorption band (Fig. 2f).

**Table 2:** Photophysical data of **Pt1** and **Pt2** in MeCN and Pt^II^@MOFs at room temperature

	Absorption	Emission	
	*λ*_max_ [nm] (*ε* [×10^3^ dm^3^ mol^–1^ cm^–1^])	*λ*_em_ [nm] (*τ* [μs])	*Φ* [%]
**Pt1** ^*a*^	277 (34.2), 321 (6.47), 378 (8.47), 394 (sh 7.59), 430 (sh 3.07)	535 (2.1)^*b*^	36^*b*^
**Pt2** ^*a*^	271 (18.1), 280 (16.6), 291 (15.6), 317 (8.69), 346 (3.17), 372 (0.84), 387 (0.82), 406 (0.54)	449 (0.2), 470^*b*^	6^*b*^
**1b**	325, 390 (sh)	527 (1.9)^*c*^	25^*c*^
**2b**	327, 405 (sh)	520 (1.8)^*c*^	19^*c*^
**3b**	330, 400 (sh)	519 (2.5)^*c*^	23^*c*^
**4b**	316, 409 (sh)	450, 477 (4.9)^*c*^	23^*c*^
**4e**		600 (3.6)^*c*^	81^*c*^

^*a*^Pt^II^ complexes at a concentration of 2 × 10^–5^ M.

^*b*^Solutions for photophysical studies were degassed using five freeze–pump–thaw cycles.

^*c*^Measured in open air.

**Fig. 3:**
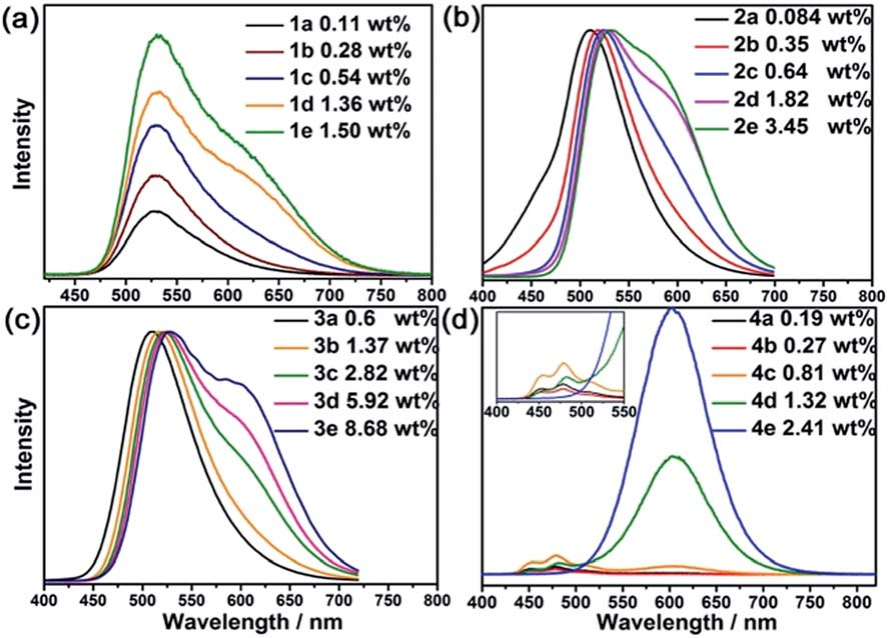
Steady-state emission spectra of Pt^II^@MOFs in open air at room temperature.

The emission properties of **1–4** were investigated. As depicted in Fig. 3, **1a** displays broad emission with *λ*_max_ at 530 nm (monomer emission). As the loading of **Pt1** increased (**1e**), another emission band at 620 nm gradually developed (aggregate emission). For the composites of **2**, **2a** displayed broad emission with *λ*_max_ at 510 nm. When the loading of **Pt1** increased (**2d** and **2e**), there was a gradual redshift of the monomer *λ*_max_ accompanied by the appearance of aggregate emission as a shoulder at 590 nm. The redshift of the monomer emission is ascribed to the intermolecular interactions of Pt^II^ complexes in the ground state.^16^ A similar redshift of the high energy emission band by ∼20 nm was also found for **3**, the framework of which contains the same organic ligand as **2**. A more distinct aggregate emission was observed for **3e**, presumably because of the higher concentration of **Pt1**, which enhances the formation of aggregate species. Similarly, **Pt1** in dilute solutions ((1–5) × 10^–5^ M, Fig. S11, ESI†) displayed intense, unstructured emission with *λ*_max_ at 530 nm and an additional emission band at 615 nm in concentrated solutions (10^–4^–10^–3^ M). For the composites of **4**, a vibronic structured blue emission band^17^ with *λ*_max_ at 450 and 470 nm was found for **4a**, whereas an additional broad emission band at 600 nm became apparent in **4d**, which has a much higher **Pt2** loading. For **4e**, which has the highest **Pt2** loading (2.41 wt%), only aggregate emission was observed and the emission quantum yield of **4e** was 81%. For comparison, **Pt2** in dilute solution (Fig. S11b, ESI†) ((1–5) × 10^–5^ M) showed vibronic structured emission with *λ*_max_ at 449 and 470 nm, whereas a broad emission peak at 593 nm was observed in concentrated solutions (10^–4^–10^–3^ M). In contrast to composite **4e**, the monomer emission of **Pt2** could still be observed in solution, even at a concentration of 1 × 10^–3^ M.

**Fig. 4:**
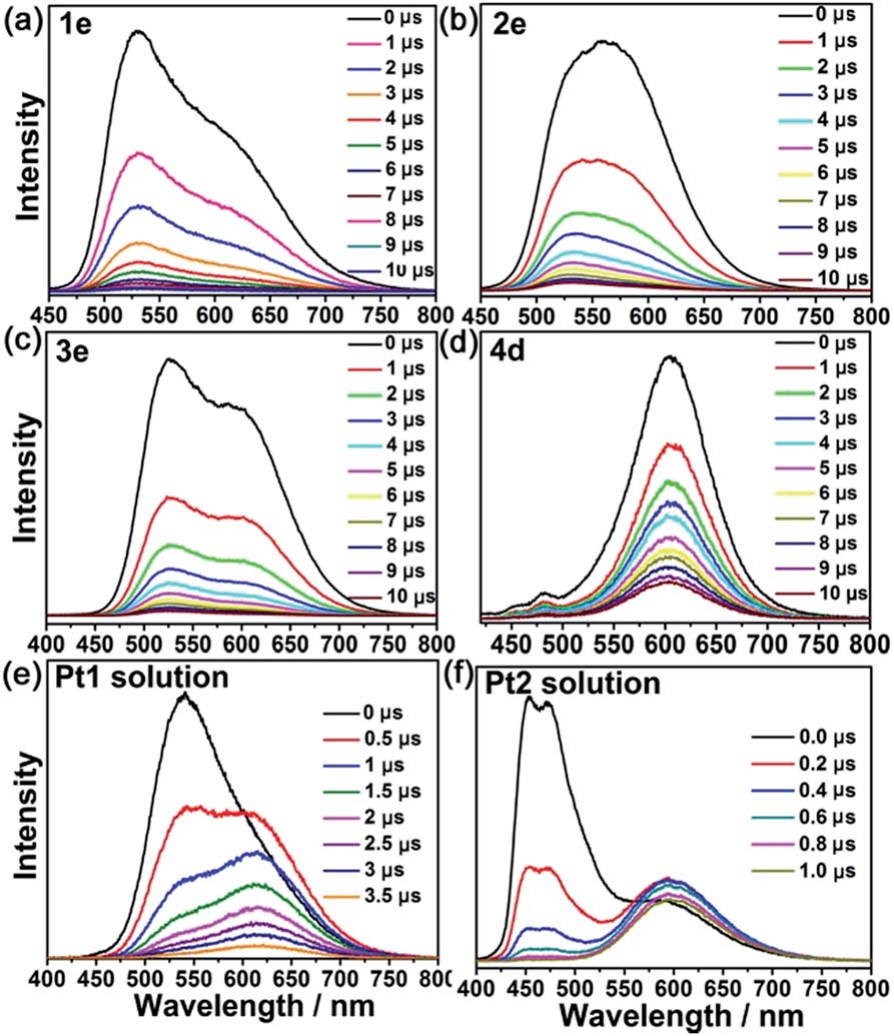
(a)–(d): Time-resolved emission spectra of Pt^II^@MOFs in open air at room temperature. (e) and (f) Time-resolved emission spectra of the Pt^II^ complexes ([**Pt1**] = 5 × 10^–4^ M; [**Pt2**] = 1 × 10^–3^ M) in degassed MeCN solutions.

Nanosecond time-resolved emission spectra of both **Pt1** (5 × 10^–4^ M) and **Pt2** (1 × 10^–3^ M) in MeCN (Fig. 4) exhibited gradually developing emission at ∼600 nm in addition to prompt phosphorescence at 530 nm for **Pt1** and 449–470 nm for **Pt2** (Fig. 4e). The growth and subsequent decay of this low energy (∼600 nm) emission is similar to the excimeric emission formed between the excited state and ground state of the Pt^II^ complex.^1^,^18^ However, different kinetic behavior for the low energy emission was observed in the Pt^II^@MOF composites. The aggregate emissions of **1e**, **2e**, **3e** and **4d** were instantaneously generated after laser pulse excitation, similar to their corresponding monomer emission. In **4e** (Fig. S12, ESI†), only one phosphorescence band with *λ*_max_ at 600 nm was detected, which was consistent with the result from the steady-state measurement. The nearly simultaneous decay in the beginning of the low and high energy emission bands is indicative of the absence of excimeric emission in Pt^II^@MOFs, and therefore, the aggregate emissions of Pt^II^@MOFs are proposed to originate from ground state aggregate species. Comparing the electronic absorption spectra of **1–4** with their corresponding excitation spectra (Fig. S13, ESI†), which showed vastly different excitation profiles for emission at 450–530 nm and 600–620 nm, led to the attribution of their aggregate emissions to a ^3^MMLCT excited state of ground state aggregates of the Pt^II^ complexes^19^ within the pores/channels of the MOFs. The origin of the difference in emission for the aggregated emissions of Pt^II^ in solution and in the MOFs suggests that the photophysical properties of Pt^II^ complexes could be altered by employing MOFs as host materials.

### Photo-catalysis

**Fig. 5:**
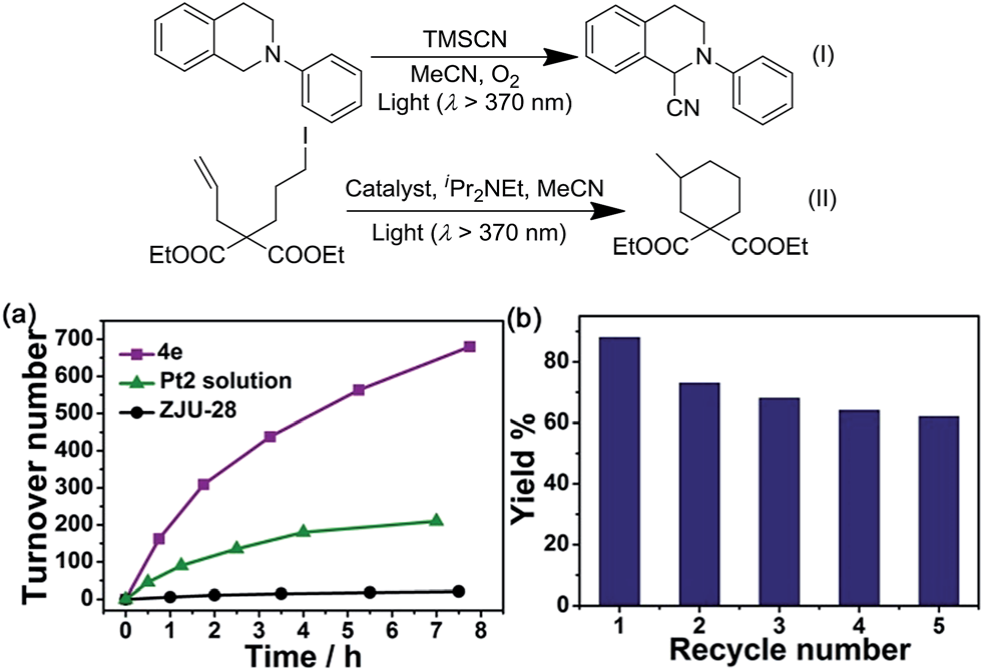
(Top) Scheme of oxidative cyanation of a tertiary amine (I) and reductive cyclization of an alkyl iodide (II). (Bottom) (a) The time course of the oxidative cyanation by **4e** (Pt2@ZJU-28), **Pt2** and ZJU-28; (b) the yield of the reaction in recycling experiments using **4e** as the catalyst.

Although several Pt^II^ complexes with high energy ^3^IL or ^3^MLCT excited states (>2.5 eV) are active catalysts for photooxidation and photoinduced aerobic C–C bond formation reactions, there have been few reports on employing complexes with low energy ^3^MMLCT excited states for such reactions.3d,^20^ Composite **4e**, which displays a predominant ^3^MMLCT excited state, was examined as a catalyst for the photoinduced α-cyanation of tertiary amines and reductive cyclization of alkyl iodides. These two reactions were performed in MeCN at room temperature (RT) under light irradiation (*λ* > 370 nm, Fig. 5). For the α-cyanation reaction, a product turnover number (TON) of ∼680 was achieved over 8 hours of irradiation (turnover frequency (TOF): 90.6, Table S2, ESI†) with 100% substrate conversion and 88% product yield. The catalyst could be recycled by washing with MeCN. After five cycles, the yield still reached a good value of ∼63%. No leaching of **Pt2** was observed after the photochemical reaction, using ICP-MS analysis of the recovered **4e**. When using **Pt2** (5 × 10^–4^ M) as a catalyst, the product TON was found to be ∼30% of that of **4e** under the same conditions. With ZJU-28 alone as a catalyst, the product TON was <10. For the photoinduced cyclization reaction, a TON of ∼155 for the desired product was achieved with **4e** over 10 hours (TOF: 15.5) with 99% yield; this TON was 5-fold higher than that found with **Pt2** in the corresponding homogeneous reaction.

**Fig. 6:**
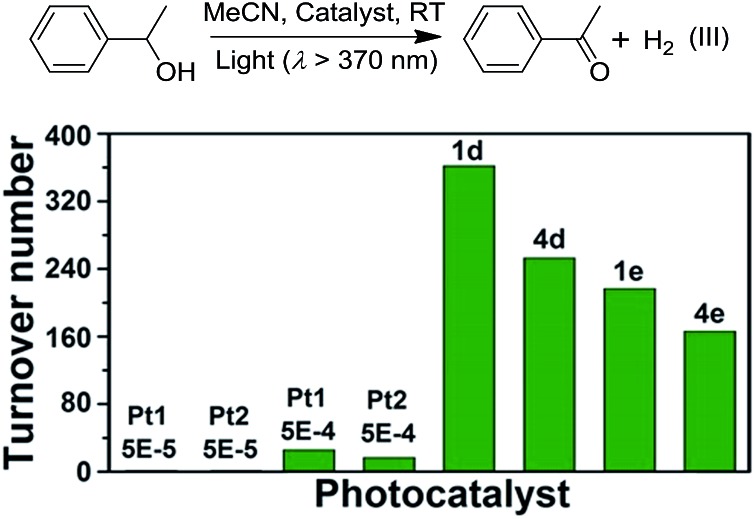
(Top) Photoinduced dehydrogenation reaction catalyzed by various catalysts (III). (Bottom) The TONs of acetophenone in reaction III using Pt complexes at different concentrations and **1d**, **4d**, **1e** and **4e** as the catalysts.

We envisage that the ^3^MMLCT excited states of the Pt^II^@MOF composites are highly reactive and can be harnessed for photoinduced C–H dehydrogenation reactions, similar to [Pt_2_(P_2_O_5_H_2_)_4_]^4–^.1*a* This type of reaction proceeds *via* inner-sphere atom abstraction by triplet excited species with vacant coordination site(s).^21^ The photocatalytic activity of Pt^II^@MOFs towards the conversion of 1-phenylethanol, benzyl alcohol, isopropanol (IPA) and cyclohexene to the corresponding ketone, aldehyde and cyclohexane was evaluated using **1** and **4** as the catalysts (Fig. 6 and Table 3) and MeCN as the solvent under a N_2_ atmosphere and at RT.

**Table 3:** Photoinduced dehydrogenation of alcohols and cyclohexene^*a*^

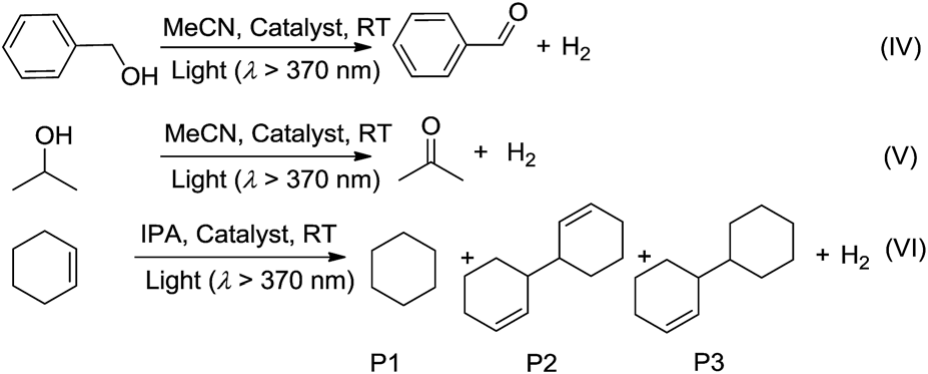						
Catalyst		TONs of reaction IV	TONs of reaction V	TONs of reaction VI^*b*^		
				P1	P2	P3
1d		47.5	10.1	23.6	4.2	1.1
1e		30.1	16.6	31.2	7.5	1.5
4d		38.2	6.1	11.3	1.3	0.4
4e		29.4	9.5	28.5	2.4	1.0
**Pt1**	5 × 10^–4^ M	10.3	—	—	—	—
	5 × 10^–5^ M	—	—	—	—	—
**Pt2**	5 × 10^–4^ M	8.3	—	—	—	—
	5 × 10^–5^ M	—	—	—	—	—
ZJU-28		—	—	—	—	—

^*a*^For detailed reaction conditions, please refer to the ESI.

^*b*^The amount of acetone formed cannot be determined because of the overlap of its GC signal with that of cyclohexene.

Irradiation (*λ* > 370 nm) of an MeCN solution of 1-phenylethanol for 6 hours with **1d** or **1e** as the catalyst provided acetophenone with TONs of 363 and 216 (TOF: 60.5 and 36), respectively (Fig. 6, the TON for hydrogen was not determined due to its possible adsorption on the inner surface of the MOF materials). Similar photochemical reactions with **Pt1** at concentrations of 0.5–5 × 10^–4^ M afforded trace amounts of the product. Similarly, composites **4d** and **4e** showed superior performance compared to **Pt2** in the same photoinduced reaction. The leaching of the Pt^II^ complex from the composites was not observed after photolysis according to ICP-MS analysis (Table S1, ESI†). After catalysis, no obvious changes in the PXRD patterns were detected (Fig. S14 and S15, ESI†). The control experiment using pure ZJU-28 as the catalyst did not show obvious product formation. As composites **1d**, **1e**, **4d** and **4e** show a predominant ^3^MMLCT excited state upon photoexcitation, the photo-catalysis results suggest that the ^3^MMLCT excited states in Pt^II^@MOF materials are responsible for the observed photocatalytic C–H bond dehydrogenation reactions. When 1-phenylethanol was replaced with benzyl alcohol, a TON of 47.5 for benzaldehyde was produced using **1d**, which is ∼5-fold higher than that obtained in **Pt1** solution at 5 × 10^–4^ M (Table 3). A negligible amount of product was detected when a low concentration (5 × 10^–5^ M) of **Pt1** was used. This divergence in reactivity was also observed between **4d** and **Pt2** in solution. Pt^II^@MOFs also reacted with IPA to furnish TONs of 6.1–16.6 of acetone upon light irradiation for 12 hours (Table 3). However, an MeCN solution of **Pt1** or **Pt2** did not show obvious acetone formation under similar conditions. Furthermore, after a mixture of IPA and cyclohexene was irradiated in the presence of **1d** or **1e** for 6 hours, cyclohexane was furnished with TONs of 23.6 and 31.2 (Table 3), respectively. The homocoupling product of the as-formed cyclohexenyl radicals (P2) and their partially hydrogenated derivative (P3) were also detected in the reaction mixture. The formation of cyclohexane is proposed to originate from the hydrogenation of cyclohexene by an *in situ*-generated Pt–H species, which might be formed from the abstraction of the allylic C–H atom of cyclohexene or from the reaction with IPA.1*a* To elucidate the origin of the hydrogen atoms, deuterated (d_8_) IPA was used in the reaction. Signals with a *m*/*z* of 84, 162 and 164, which correspond to non-deuterated P1, P2 and P3, respectively, could still be detected as the sole products by GC-MS, thereby excluding the possibility of IPA serving as the H-atom source. Notably, when the same reaction was conducted with **Pt1** or **Pt2** as the catalyst in homogeneous solution, cyclohexane was not detected. Pt^II^@MOFs can also catalyze the photoinduced dehydrogenation of indoline and 1,2,3,4-tetrahydroquinoline (Table 4). Irradiation of an MeCN solution containing indoline and **1d** or **4d** produced 1H-indole (P4) with TONs of 58.6 and 44.3, respectively, which were approximately 6-fold and 17-fold higher than those obtained using **Pt1** or **Pt2** at a concentration of 5 × 10^–4^ M as the photocatalyst. When the concentration of **Pt1** or **Pt2** in the homogeneous reaction was reduced to 5 × 10^–5^ M, only a trace amount of 1H-indole was detected. For the dehydrogenation reaction of 1,2,3,4-tetrahydroquinoline, TONs of 25–27.2 of 3,4-dihydroquinoline (P5) and TONs of 8.3–12.3 of quinoline (P6) were produced using **1d** and **4d** (Table 4).

**Table 4:** Photoinduced dehydrogenation of indoline and 1,2,3,4-tetrahydroquinoline

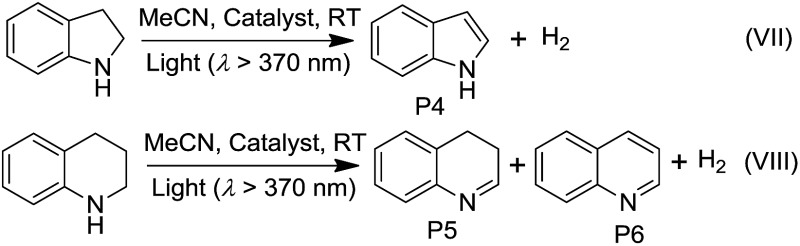			
Catalyst	TONs of reaction VII	TONs of reaction VIII	
	P4	P5	P6
**1d** ^*a*^	58.6	27.2	12.3
**4d** ^*a*^	44.3	25.0	8.3
**Pt1** ^*b*^	10.1	4.1	<1
**Pt2** ^*b*^	2.5	3.3	1.0
ZJU-28^*a*^	—	—	—

^*a*^MOF material (10 mg), substrate (0.1 mmol), MeCN (2 mL) and light (*λ* > 370 nm).

^*b*^5 × 10^–4^ M of Pt^II^ complex was used instead of the MOF material.

Pt^II^@MOFs also catalyzed the photoinduced dehydrogenative cyclization of *o*-aminobenzamide with benzyl alcohol at RT. 2-Phenylquinazolin-4(3*H*)-one (P7) was obtained with TONs of 14.9 and 9.4 using **1d** and **4d** as photocatalysts, respectively (Table 5). In contrast to the heterogeneous catalyst, **Pt1** and **Pt2** at a concentration of 5 × 10^–4^ M in MeCN did not show catalytic activity in this reaction.

**Table 5:** Dehydrogenative coupling of *o*-aminobenzamide with benzyl alcohol

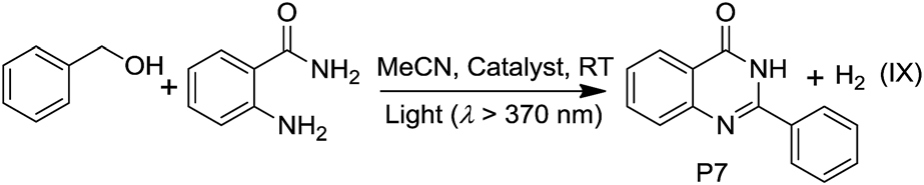	
Catalyst	TONs of reaction IX (P7)
**1d** ^*a*^	14.9
**4d** ^*a*^	9.4
**Pt1** ^*b*^	—
**Pt2** ^*b*^	—
ZJU-28^*a*^	—

^*a*^MOF material (10 mg), *o*-aminobenzamide (0.1 mmol) and benzyl alcohol (0.1 mmol), MeCN (2 mL), light (*λ* > 370 nm).

^*b*^5 × 10^–4^ M of Pt^II^ complex was used instead of the MOF material.

The improved performances of Pt^II^@MOF catalysts such as **1d** and **4d** over that of **Pt1** and **Pt2**, respectively, in the photo-catalysis described above reveals the beneficial effects of encapsulation of the Pt^II^ complexes in the pores of the MOF hosts. For the photo-induced catalytic α-cyanation of tertiary amines (reaction I) and reductive cyclization of alkyl iodide (reaction II), which are proposed to proceed *via* singlet oxygen^11^,^22^ and outer-sphere electron transfer^23^ pathways, respectively, the higher activity of Pt^II^@MOF catalysts relative to Pt^II^ complexes is reminiscent of the better catalytic activity of Au^III^@MOF than that of Au^III^ complexes for the two reactions.^11^ In the cases of the other photo-induced catalytic reactions studied in this work, that is, photo-induced dehydrogenation reactions, the enhancement of the catalytic activity upon formation of Pt^II^@MOFs could be ascribed to the aggregation of the Pt^II^ complexes in the pores of the MOF hosts resulting in the ^3^MMLCT excited state. Based on the mechanism proposed for the [Pt_2_(μ-P_2_O_5_H_2_)_4_]^4–^ system,1*a* and considering the observed hydrogenated by-products in the cyclohexene reaction, a mechanism involving light irradiation generating the ^3^MMLCT excited state species (Pt–Pt)* which abstracts a hydrogen atom from, for example, the α-C–H or allylic C–H bond of the alcohol, indoline, 1,2,3,4-tetrahydroquinoline or cyclohexene substrate to form a H–(Pt–Pt) species, is proposed for the Pt^II^@MOF system; the H–(Pt–Pt) species may further abstract a hydrogen atom, forming H_2_(Pt–Pt) and the dehydrogenation product, and H_2_ is eliminated from the H_2_(Pt–Pt) species to regenerate (Pt–Pt). The formation of a reactive H–(Pt–Pt) species could be inferred from the formation of cyclohexane from cyclohexene. For the dehydrogenative coupling reaction (Table 5), the photo-induced dehydrogenation of benzyl alcohol catalyzed by Pt^II^@MOF would generate benzaldehyde, which undergoes a condensation reaction with *o*-aminobenzamide, followed by an intramolecular nucleophilic attack on the carbon of the C=N imine bond by NH_2_ of the amide group, to give a 2-phenyl-2,3-dihydroquinazolin-4(1*H*)-one intermediate; dehydrogenation of this intermediate by Pt^II^@MOF gives the final product.

## Conclusions

A series of Pt^II^@MOFs composites displaying strong matrix-dependent phosphorescence were prepared *via* a cation exchange method. The cages and nanotubes of the MOFs function as concentrators for the Pt^II^ complexes and induce aggregation inside the MOFs, leading to ^3^MMLCT emission. With the diradical character of the ^3^MMLCT excited state, the Pt^II^@MOFs showed superior performance in photoinduced C–C bond formation, dehydrogenation and dehydrogenative cyclization reactions compared to the corresponding Pt^II^ complexes in solution. This simple approach for preparing highly photocatalytically active MOF composites offers a new entryway to new classes of phosphorescent and heterogeneous photofunctional materials with useful applications.

## Conflicts of interest

There are no conflicts to declare.

## Supplementary Material

Supplementary information
