# Supplementary material for: Metal–organic framework composites with luminescent pincer platinum(ii) complexes: 3MMLCT emission and photoinduced dehydrogenation catalysis
Source: Chem Sci. 2018 Feb 1;9(8):2357–64. doi: 10.1039/c7sc04528j (PMC5897847; doi:10.1039/c7sc04528j)
Supplement: Supplementary file 1 [file SC-009-C7SC04528J-s001.pdf]

Electronic Supplementary Material (ESI) for Chemical Science.
This journal is © The Royal Society of Chemistry 2018

Electronic Supplementary Information

Table of contents

**Experimental Section**	S2
Materials and methods	S2
Synthesis of **Pt1** and **Pt2** complexes	S2
Synthesis of Pt^{II}@MOFs composites	S3
Simulation of Pt^{II} complex adsorbed in the pores of host MOF	S3
General procedure for photochemical reactions	S4
**References**	S7
**Table S1** The concentration of Pt^{II} complexes in Pt^{II}@MOFs **1d** and **4d** after photo-catalysis determined by ICP-MS.	S8
**Table S2** Mole numbers of product, TONs, and TOFs for reactions I–IX.	S9
**Fig. S1** Photographs of ZJU-28, MOF1, MOF2, **1e**, **2e**, **3e** and **4e**.	S10
**Fig. S2** PXRD patterns of host MOFs and Pt^{II}@MOFs composites.	S11
**Fig. S3** Optical microscopy images under irradiation by UV light (365 nm) of a freshly prepared Pt1@MOF1 crystal and after this crystal was split by a needle.	S12
**Fig. S4** Scanning electron microscope (SEM) image and energy dispersive X-ray (EDX) elemental mapping (In and Pt) of split crystals of **1a**–**1e**.	S13
**Fig. S5** Scanning electron microscope (SEM) image and energy dispersive X-ray (EDX) elemental mapping (Zn and Pt) of split crystals of **2a**–**2e**.	S14
**Fig. S6** Scanning electron microscope (SEM) image and energy dispersive X-ray (EDX) elemental mapping (Zn and Pt) of split crystals of **3a**–**3e**.	S15
**Fig. S7** Scanning electron microscope (SEM) image and energy dispersive X-ray (EDX) elemental mapping (In and Pt) of split crystals of **4a**–**4e**.	S16
**Fig. S8** The location site of **Pt1** in MOF2.	S17
**Fig. S9** Nitrogen sorption isotherms of MOF1 and **2e**.	S18
**Fig. S10** Electronic absorption spectra of MOFs.	S19
**Fig. S11** Emission spectra of **Pt1** and **Pt2** in degassed MeCN upon excitation at 380 nm.	S20
**Fig. S12** Time-resolved emission spectra of **4e** in open air at room temperature.	S21
**Fig. S13** Excitation spectra of Pt^{II}@MOFs at specified emission wavelengths in open air at room temperature.	S22
**Fig. S14** PXRD patterns of as-synthesized ZJU-28, **1d**, and **1d** after catalysis for reactions III-VIII.	S23
**Fig. S15** PXRD patterns of as-synthesized ZJU-28, **4d**, and **4d** after catalysis for reactions III-IX.	S24

Experimental Section

Materials and methods: All reagents and solvents for syntheses were purchased from commercial sources and used as received, unless otherwise indicated. ZJU-28,[1] MOF1 [2] and MOF2 [3] and Pt^{II} complexes[4] were synthesized according to the literature procedures. PXRD patterns were recorded on a Siemens D5005 diffractometer with Cu Kα (λ = 1.5418 Å) radiation in the range of 3−50°. Elemental analyses (C, H, and N) were performed on a Perkin-Elmer 240C elemental analyzer. Steady-state emission spectra were recorded on a SPEX 1681 Fluorolog-3 spectrophotometer. UV/Vis absorption spectra for **Pt1** and **Pt2** in MeCN solutions were recorded on a Hewlett-Packard 8453 diode array spectrophotometer, and those of MOF materials were obtained on U-3010 spectrophotometer (Hitachi, Japan). Emission lifetime measurements were performed on a Quanta Ray GCR 150-10 pulsed Nd:YAG laser system. Errors for λ values (±1 nm), and τ (±10%) are estimated. Solutions for photophysical studies were degassed by using a high vacuum line in a two-compartment cell with five freeze-pump-thaw cycles. The photoluminescence quantum yields were measured using Hamamatsu multichannel analyzer c10027. Nuclear magnetic resonance spectra were recorded on Bruker DPX-400 or Avance 600 FT-NMR spectrometer with chemical shifts (in ppm) relative to tetramethylsilane or non-deuterated solvent residual. Time-resolved emission spectra were recorded on a LP920-KS Laser Flash Photolysis Spectrometer (Edinburgh Instruments Ltd, Livingston, UK). The excitation source was 355 nm output from a Nd:YAG laser. An Agilent 7890A GC with both flame ionization detector (FID; with either a HP-5 column or HP-FFAP column) and thermal conductivity detector (TCD; with a 5 Å molecular sieve column and argon as carrier gas and reference gas) was used for analysis of products in photochemical reactions. Analysis with the use of GC-MS was done on an Agilent 7890B GC system, with 5977A MSD, and a HP-5 column.

Synthesis of Pt1

[Pt(C^N^C)(C≡CC$_6$H$_5$)]PF$_6$:[4] A mixture of [Pt(C^N^C)Cl]OTf (55 mg, 0.08 mmol) and phenylacetylide ligand (0.10 mmol) in MeCN (40 mL) was degassed by bubbling N_2 through the solution. Triethylamine (0.5 mL) and CuI (2 mg, 0.01 mmol) were added to the solution. The resultant mixture was stirred at room temperature for 12 h. Upon removal of solvent, the crude product was dissolved in a saturated NH_4PF_6 solution in MeCN (20 mL) and then filtered through Celite. The orange solution obtained was evaporated to dryness and the product was purified by chromatography on a neutral Al_2O_3 column using CH_2Cl_2/MeCN (2:1 v/v) as eluent. Yield: 40 mg, 65%. ^{1}H NMR (400 MHz, MeCN-d_3): δ 0.88 (t, *J* = 7.4 Hz, 6H), 1.33 (m, 4H), 1.88 (m, 4H), 4.65 (t, *J* = 7.2 Hz, 4H), 7.20–7.38 (m, 7H), 7.62 (m, 2H), 7.85 (d, *J* = 2.0 Hz, 2H), 8.32 (t, *J* = 8.2 Hz, 1H). ^{19}F{^{1}H} NMR (376 MHz, MeCN-d_3): δ -72.0, -73.9. IR (KBr): ν(C≡C, w) 2116 cm^{-1}. FAB-MS (+ve, *m*/*z*): 619 [M$^+$]. Elemental analyses for $C_{27}H_{30}N_5PtPF_6 \cdot H_2O$: C, 41.44; H, 4.12; N, 8.95. Found: C, 41.66; H, 4.14; N, 9.16.

Synthesis of Pt2

[Pt(C^N^C)(CN)]PF$_6$: A mixture of [Pt(C^N^C)Cl]OTf (60 mg, 0.09 mmol) and AgOTf (28 mg,

0.11 mmol) in MeCN (40 mL) was heated under reflux for 12 h. The solution was filtered and then mixed with a solution of NEt_4CN in MeCN (10 mL). The resultant mixture was stirred at room temperature for 12 h. Afterward, the solution was concentrated to about 2 mL and added to an aqueous solution saturated with NH_4PF_6 to give the desired complex as a solid. Yield: 35 mg, 58%. ^{1}H NMR (600 MHz, MeCN-d_3): δ 0.96 (t, J = 7.4 Hz, 6H), 1.39–1.46 (m, 4H), 1.85–1.90 (m, 4H), 4.45 (t, J = 7.4 Hz, 4H), 7.37 (d, J = 2.2 Hz, 2H), 7.62 (d, J = 8.3 Hz, 2H), 7.84 (d, J = 2.2 Hz, 2H), 8.34 (t, J = 8.3 Hz, 1H). ^{13}C NMR (150 MHz, MeCN-d_3): δ 13.9, 20.1, 33.9, 52.3, 108.9, 112.4, 120.0, 124.5, 148.3, 152.8, 167.7. ^{19}F{^{1}H} NMR (376 MHz, MeCN-d_3): δ -72.0, -73.9. IR (KBr): ν(C≡N, w) 2139 cm^{-1}. FAB-MS (+ve, m/z): 544 [M$^+$]. Elemental analyses for $C_{20}H_{25}N_6PtPF_6$: C, 34.84; H, 3.65; N, 12.19. Found: C, 35.02; H, 4.05; N, 12.02.

Synthesis of Pt^{II}@MOFs composites

Synthesis of 1a-1e composites: ZJU-28 (10 mg) was suspended in 2 mL of DMF solutions containing 2.5 × 10^{-5}, 5 × 10^{-5}, 1 × 10^{-4}, 5 × 10^{-4}, 1 × 10^{-3} M (**1a-1e**) of **Pt1** complexes, respectively, with shaking in 4-mL sealed glass vials. After 3 days, the immersed samples were taken out and washed with DMF (~4 × 10 mL) until the washings were colorless (to remove residual **Pt1** complex on the surface). The concentrations of encapsulated **Pt1** complexes in composite materials were measured by ICP-MS and the results are shown in Table 1.

Synthesis of 2a-2e composites: MOF1 (10 mg) was suspended in 2 mL of DMF solutions containing 2.5 × 10^{-5}, 5 × 10^{-5}, 1 × 10^{-4}, 5 × 10^{-4}, 1 × 10^{-3} M (**2a-2e**) of **Pt1** complexes, respectively, with shaking in 4-mL sealed glass vials. After 3 days, the immersed samples were taken out and washed with DMF (~4 × 10 mL) until the washings were colorless (to remove residual **Pt1** complex on the surface). The concentrations of encapsulated **Pt1** complexes in composite materials were measured by ICP-MS and the results are shown in Table 1.

Synthesis of 3a-3e composites: MOF2 (10 mg) was suspended in 2 mL of DMF solutions containing 2.5 × 10^{-5}, 5 × 10^{-5}, 1 × 10^{-4}, 5 × 10^{-4}, 1 × 10^{-3} M (**3a-3e**) of **Pt1** complexes, respectively, with shaking in 4-mL sealed glass vials. After 3 days, the immersed samples were taken out and washed with DMF (~4 × 10 mL) until the washings were colorless (to remove residual **Pt1** complex on the surface). The concentrations of encapsulated **Pt1** complexes in composite materials were measured by ICP-MS and the results are shown in Table 1.

Synthesis of 4a-4e composites: ZJU-28 (10 mg) was suspended in 2 mL of MeCN solutions containing 2.5 × 10^{-5}, 5 × 10^{-5}, 1 × 10^{-4}, 5 × 10^{-4}, 1 × 10^{-3} M (**4a-4e**) of **Pt2** complexes, respectively, with shaking in 4-mL sealed glass vials. After 3 days, the immersed samples were taken out and washed with MeCN (~4 × 10 mL) until the washings were colorless (to remove residual **Pt2** complex on the surface). The concentrations of encapsulated **Pt2** complexes in composite materials were measured by ICP-MS and the results are shown in Table 1.

Simulation of Pt^{II} complex adsorbed in the pores of host MOF

All of the DFT calculations were performed by using Vienna ab initio simulation package (VASP).[5,6] The exchange–correlation energy was treated based on the generalized gradient

approximation (GGA) in the scheme of Perdew–Burke–Ernzerhof (PBE).[7] The core–electron interactions were described by Projector–augmented–wave (PAW) pseudopotentials.[8] To describe the van der Waals (vdW) interaction in the systems properly, DFT with the empirical dispersion correction (DFT–D) method was applied due to its good description of long–range vdW interactions.[9] A Monkhorst–Pack k–point mesh of 1 × 1 × 1 was used to perform the geometric optimizations. The energy cutoff was set to be 420 eV and all atoms were fully relaxed until the total energy converge to less than 10^{-4} eV. The calculated model is performed in a single cell whose lattice parameters are as follows: $a = b = 22.915$ Å, $c = 32.892$ Å and $\alpha = \beta = \gamma = 90°$. To describe the interaction between adsorbates and MOFs, the adsorption energy was calculated as follows:

$$E_{ads} = E_{MOF}^{Pt} - E^{Pt} - E_{MOF}$$

where E_{MOF}^{Pt} is the total energy of MOF with adsorbate, E_{MOF} is the total energy of the MOF and E^{Pt} is the energy of the adsorbates, respectively. The Pt was optimized in a three-dimensional box of $a = b = c = 30$ Å.

General procedure for photochemical reactions

Detailed procedures for the photochemical reactions I-IX are described in the following. Control experiments, including those described in text and also dark controls, were performed under similar conditions, which did not result in obvious product formation. After catalytic reactions, the contents of Pt^{II} complexes in Pt^{II}@MOF catalysts and the PXRD patterns of these catalysts were checked; examples of which are depicted in Table S1, Fig. S14 and Fig. S15. The reaction products were identified by comparing with the spectral data reported in the literature[10] and/or by GC-MS analysis.

(1) Oxidative cyanation of tertiary amine by 4e, Pt2 and ZJU-28 (Reaction I)

The catalyst (**4e** (10 mg), **Pt2** (1×10^{-6} mol), or ZJU-28 (10 mg)) was added into a test tube, and then a mixture of 2-phenyl-1,2,3,4-tetrahydroisoquinoline (0.053 mmol, 11 mg) and trimethylsilanecarbonitrile (0.106 mmol, 13 μL) in MeCN (1.6 mL) was added. The reaction mixture was bubbled with solvent-saturated oxygen gas throughout the experiment (1 atm) and irradiated at $\lambda > 370$ nm (with a 300 W Xenon lamp as the light source) at room temperature. Every 2 h, equivalent amounts of substrate and TMSCN were added into the solution. The product yield (based on conversion) was determined by ^{1}H NMR spectroscopic analysis.[11] For recycling experiments, **4e** was taken out after catalysis via centrifugation, then washed with MeCN and added into MeCN solution (1.6 mL) containing 2-phenyl-1,2,3,4-tetrahydroisoquinoline (0.053 mmol, 11 mg) and trimethylsilanecarbonitrile (0.106 mmol, 13 μL). The reaction mixture was bubbled with oxygen and irradiated under the same condition.

(2) Reductive cyclization of alkyl iodide using 4e and Pt2 as catalysts (Reaction II)

The catalyst (**4e** (10 mg), **Pt2** (1×10^{-6} mol), or ZJU-28 (10 mg)) was added into a test tube. A mixture of diethyl 2-allyl-2-(3-iodopropyl)malonate (0.1 mmol) and *N*,*N*-diisopropylethylamine (0.2 mmol) in MeCN (2 mL) was added into the test tube. After degassing by bubbling with argon,

the reaction mixture was irradiated at λ > 370 nm (with a 300 W Xenon lamp as the light source) at room temperature. The product yield was determined by ^{1}H NMR spectroscopy using 4,4′-dimethyl-2,2′-bipyridine as internal standard.[12]

(3) Photo-induced hydrogen atom abstraction from C−H bonds by Pt^{II}@MOFs, ZJU-28, Pt complexes solution (Reaction III-VIII)

- For Reaction III using 1-phenylethanol as substrate:

The catalyst (Pt^{II}@MOFs (10 mg), Pt^{II} complexes (5×10^{-5} or 5×10^{-4} M), or ZJU-28 (10 mg)) was added into a test tube, and then a mixture of 1-phenylethanol (2 mL) and MeCN (2 mL) was added into the tube. The reaction mixture was bubbled with N_2 for 5-10 min in the dark and then irradiated at λ > 370 nm with a 300 W xenon lamp as the light source at room temperature. After an irradiation time of 6 h, 200 μL of the headspace of the test tube were taken out by a Pressure-Lock syringe and injected into the GC with TCD to test for hydrogen production. The volume of the hydrogen produced was calculated by comparing the integrated area of the signals of hydrogen and nitrogen gas with a calibration curve. 6 μL of the solution were taken out and injected into the GC with FID and a HP-FFAP column to quantify the organic products.

- For Reaction IV using benzyl alcohol as substrate:

The catalyst (Pt^{II}@MOFs (10 mg), Pt^{II} complexes (5×10^{-5} or 5×10^{-4} M), or ZJU-28 (10 mg)) was added to a test tube, and then a mixture of benzyl alcohol (2 mL) and MeCN (2 mL) was added into the test tube. The reaction mixture was bubbled with N_2 for 5-10 min in the dark and then irradiated at λ > 370 nm with a 300 W xenon lamp as the light source at room temperature. After an irradiation time of 6 h, 200 μL of the headspace of the test tube was taken out by a Pressure-Lock syringe and injected into the GC with TCD to test for hydrogen production. 6 μL of the solution were taken out and injected into the GC with FID and a HP-FFAP column to quantify the organic products.

- For Reaction V using isopropanol as substrate:

The catalyst (Pt^{II}@MOFs (10 mg), Pt^{II} complexes (5×10^{-5} or 5×10^{-4} M), or ZJU-28 (10 mg)) was added into a test tube, and then a mixture of isopropanol (2 mL) and MeCN (2 mL) was added into the test tube. The reaction mixture was bubbled with N_2 for 5-10 min in the dark and then irradiated at λ > 370 nm with a 300 W xenon lamp as the light source at room temperature. After an irradiation time of 12 h, 200 μL of the headspace of the test tube were taken out by a Pressure-Lock syringe and injected into the GC with TCD to test for hydrogen production. 6 μL of the solution were taken out and injected into the GC with FID and a HP-FFAP column to quantify the organic products.

- For Reaction VI using cyclohexene as substrate:

The catalyst (Pt^{II}@MOFs (10 mg), Pt^{II} complexes (5×10^{-4} M), or ZJU-28 (10 mg)) was added into a test tube, and then a mixture of cyclohexene (1.5 mL) and isopropanol (1.5 mL) was added into the test tube. The reaction mixture was bubbled with N_2 for 5-10 min in the dark and then irradiated at λ > 370 nm with a 300 W xenon lamp as the light source at room temperature. After an irradiation time of 14 h, 200 μL of the headspace of the test tube were taken out by a Pressure-Lock syringe and injected into the GC with TCD to test for hydrogen production. 6 μL of the solution were taken out and injected into the GC with FID and a HP-5 column to quantify the

organic products. The isotopic labelling experiment was carried out under similar condition except that isopropanol was replaced with deuterated (d_8) isopropanol. The products were detected by GC-MS.

- For Reaction VII using indoline as substrate:

The catalyst (Pt^{II}@MOFs (10 mg), or Pt^{II} complexes (5×10^{-4} M), or ZJU-28 (10 mg)) was added to a tube containing indoline (0.1 mmol) in MeCN (2 mL). The reaction mixture was bubbled with N_2 for 5-10 min in the dark and then irradiated at $\lambda > 370$ nm with a 300 W xenon lamp as the light source at room temperature. After an irradiation time of 6 h, 200 μL of the headspace of the test tube were taken out by a Pressure-Lock syringe and injected into the GC with TCD to test for hydrogen production. 6 μL of the solution were taken out and injected into the GC with FID and a HP-5 column to quantify the organic products.

- For Reaction VIII using 1,2,3,4-tetrahydroquinoline as substrate:

The catalyst (Pt^{II}@MOFs (10 mg), Pt^{II} complexes (5×10^{-4} M), or ZJU-28 (10 mg)) was added into a test tube, and then 1,2,3,4-tetrahydroquinoline (0.1 mmol) in MeCN (2 mL) was added into the test tube. The reaction mixture was bubbled with N_2 for 5-10 min in the dark and then irradiated at $\lambda > 370$ nm with a 300 W xenon lamp as the light source at room temperature. After an irradiation time of 6 h, 200 μL of the headspace of the test tube were taken out by a Pressure-Lock syringe and injected into the GC with TCD to test for hydrogen production. 6 μL of the solution were taken out and injected into a GC with FID and a HP-5 column to quantify the organic products.

(4) Dehydrogenative coupling of *o*-aminobenzamide with benzyl alcohol (Reaction IX)

The catalyst (Pt^{II}@MOFs (10 mg), Pt^{II} complexes (5×10^{-4} M), or ZJU-28 (10 mg)) was added in a test tube, and then *o*-aminobenzamide (0.1 mmol) with benzyl alcohol (0.1 mmol) in MeCN (2 mL) was added into the test tube. The reaction mixture was bubbled with N_2 for 5-10 min in the dark and then irradiated at $\lambda > 370$ nm with a 300 W xenon lamp as the light source at room temperature. After an irradiation time of 12 h, 200 μL of the headspace of the test tube were taken out by a Pressure-Lock syringe and injected into the GC with TCD to test for hydrogen production. 6 μL of the solution were taken out and injected into the GC with FID detector and a HP-5 column to quantify the organic products.[13]

References

1 J. Yu, Y. Cui, C. Wu, Y. Yang, Z. Wang, M. O'Keeffe, B. Chen and G. Qian, *Angew. Chem. Int. Ed.*, 2012, **51**, 10542-10545.

2 C.-Y. Sun, C. Qin, C.-G. Wang, Z.-M. Su, S. Wang, X.-L. Wang, G.-S. Yang, K.-Z. Shao, Y.-Q. Lan and E.-B. Wang, *Adv. Mater.*, 2011, **23**, 5629-5632.

3 C.-Y. Sun, X.-L. Wang, C. Qin, J.-L. Jin, Z.-M. Su, P. Huang and K.-Z. Shao, *Chem. Eur. J.*, 2013, **19**, 3639-3645.

4 S. Y.-L. Leung, E. S.-H. Lam, W. H. Lam, K. M.-C. Wong, W.-T. Wong and V. W.-W. Yam, *Chem. Eur. J.*, 2013, **19**, 10360-10369.

5 G. Kresse and J. Furthmüller, *Phys. Rev. B*, 1996, **54**, 11169-11186.

6 G. Kresse and J. Furthmüller, *Comput. Mater. Sci.*, 1996, **6**, 15-50.

7 J. P. Perdew, K. Burke and M. Ernzerhof, *Phys. Rev. Lett.*, 1996, **77**, 3865-3868.

8 P.E. Blöchl, *Phys. Rev. B*, 1994, **50**, 17953-17979.

9 B. Van Troeye, M. Torrent and X. Gonze, *Phys. Rev. B*, 2016, **93**, 144304/1-144303/9.

10 (*a*) S.-I. Murahashi, T. Nakae, H. Terai and N. Komiya, *J. Am. Chem. Soc.*, 2008, **130**, 11005–11012; (*b*) G. Revol, T. McCallum, M. Morin, F. Gagosz and L. Barriault, *Angew. Chem. Int. Ed.*, 2013, **52**, 13342–13345; (*c*) S. Mohammed, R. A. Vishwakarma and S. B. Bharate, *J. Org. Chem.*, 2015, **80**, 6915–6921.

11 W.-P. To, G. S.-M. Tong, W. Lu, C. Ma, J. Liu, A. L.-F. Chow and C.-M. Che, *Angew. Chem. Int. Ed.*, 2012, **51**, 2654-2657.

12 P.-K. Chow, G. Cheng, G. S. M. Tong, W.-P. To, W.-L. Kwong, K.-H. Low, C.-C. Kwok, C. Ma and C.-M. Che, *Angew. Chem. Int. Ed.*, 2015, **54**, 2084-2089.

13 S. Parua, S. Das, R. Sikari, S. Sinha and N. D. Paul, *J. Org. Chem.*, 2017, **82**, 7165-7175.

Table S1 The concentration of Pt^{II} complexes in Pt^{II}@MOFs **1d** and **4d** after photo-catalysis determined by ICP-MS.

Reaction	Concentration of Pt^{II} complexes in composites (wt%)	
	**1d**	**4d**
III	1.33	1.29
IV	1.34	1.30
V	1.31	1.28
VI	1.29	1.27
VII	1.27	1.25
VIII	1.28	1.24
IX	1.30	1.27

Table S2 Mole numbers of product, TONs, and TOFs for reactions I–IX.[a]

Reaction	Mole number (μmol)	TON	TOF
I	292.4	680	90.6
II	66.6	155	15.5
III[a]	79.1	363	60.5
IV	10.4	47.5	7.9
V	2.2	10.1	0.84
VI	6.2**[b]**	28.6**[b]**	2.0**[b]**
VII	12.8	58.6	9.4
VIII[b]	8.6 **[b]**	39.5**[b]**	6.6 **[b]**
IX	3.2	14.9	1.2

[a]The listed mole numbers, TONs, and TOFs in reaction III-IX use **1d** as catalyst; for reactions I and II, **4e** was used as catalyst. [b]Mole number, TON, and TOF each are the total value of all products. TON and TOF values were based on the content of Pt^{II} complex in the Pt^{II}@MOF catalyst assuming all the encapsulated Pt^{II} complex is active.

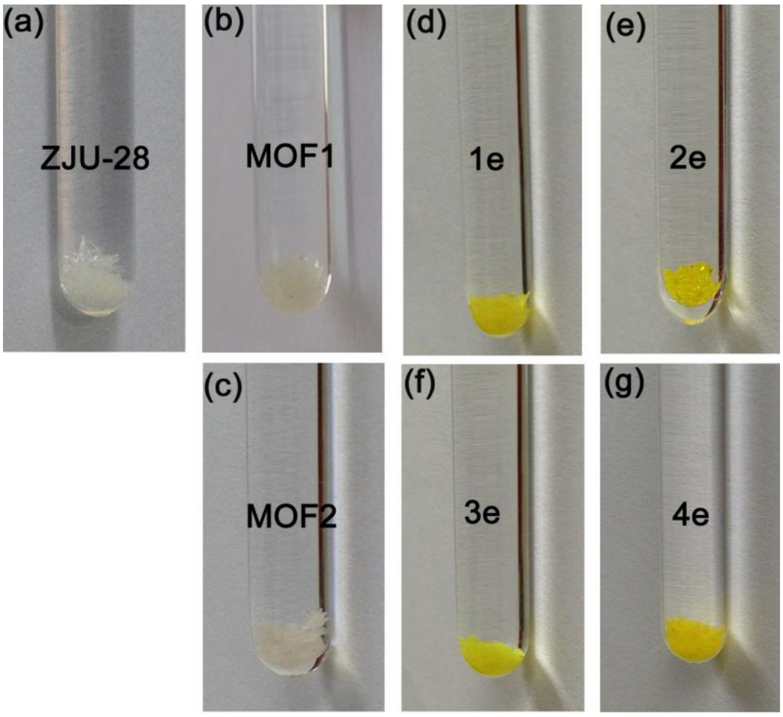

Fig. S1 Photographs of ZJU-28, MOF1, MOF2, **1e**, **2e**, **3e** and **4e**.

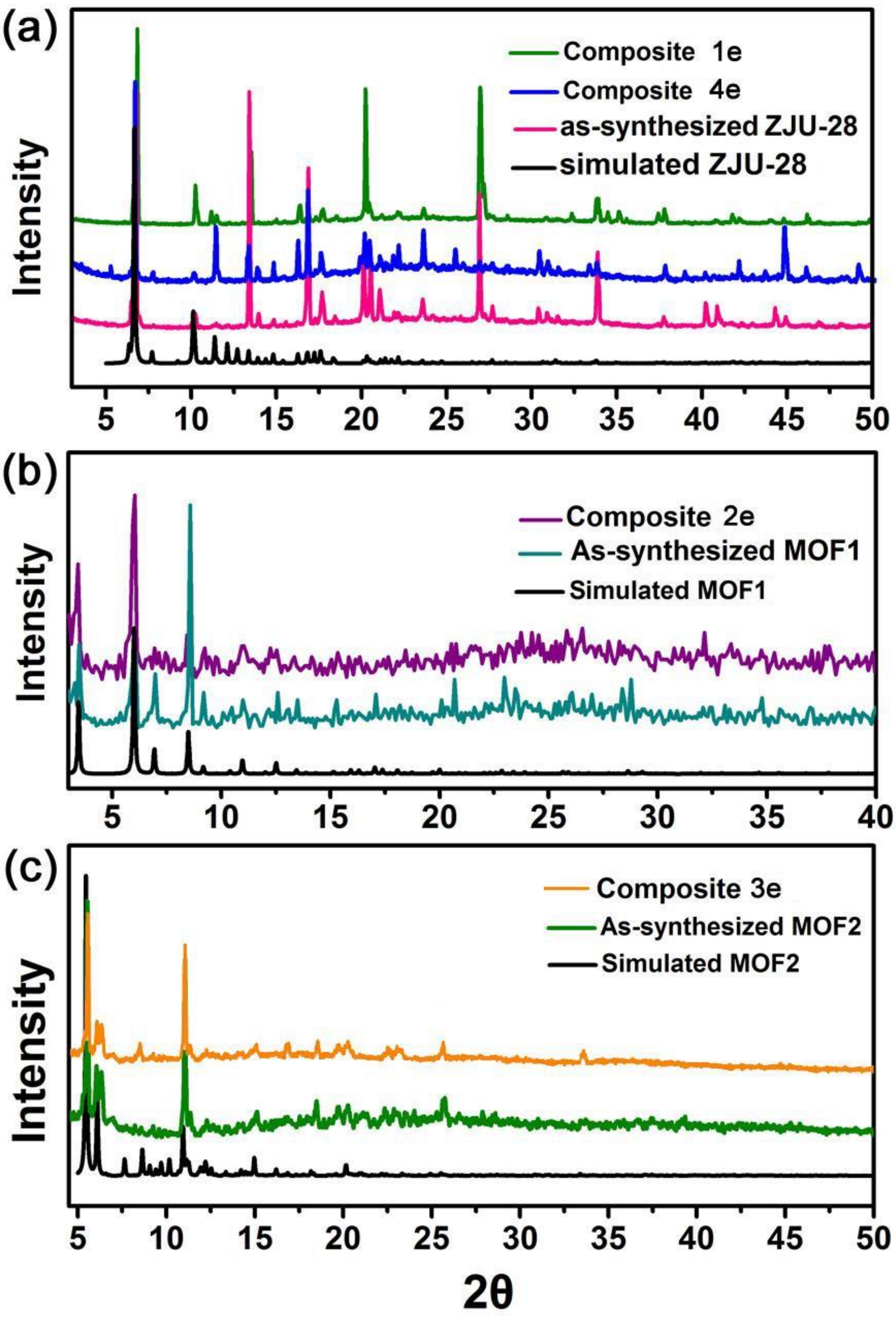

Fig. S2 PXRD patterns of ZJU-28, MOF1, MOF2, and Pt^{II}@MOFs.

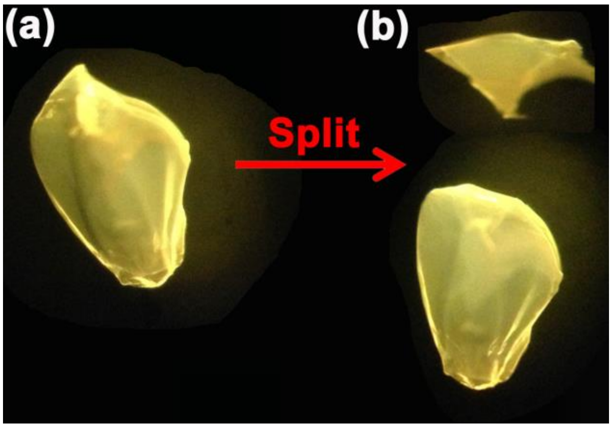

Fig. S3 Optical microscopy images under irradiation by UV light (365 nm) of (a) a freshly prepared Pt1@MOF1 crystal and (b) after this crystal was split by a needle.

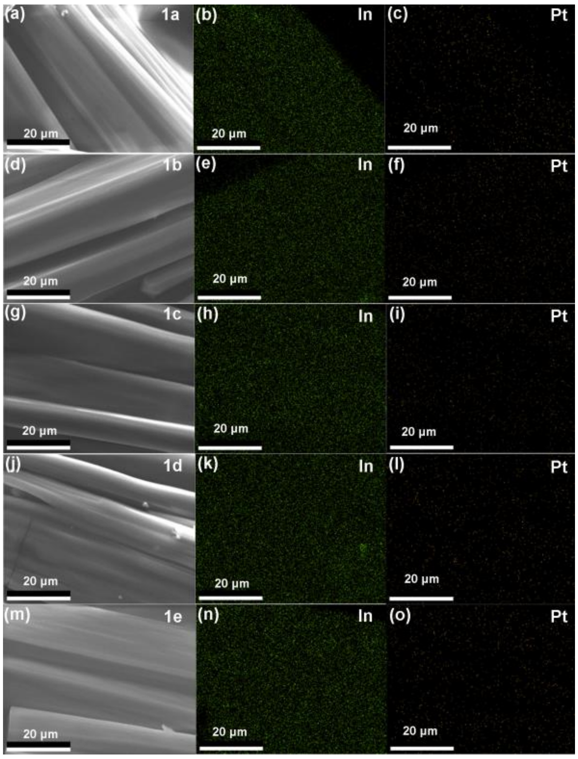

Fig. S4 Scanning electron microscope (SEM) image (a, d, g, j, m) and energy dispersive X-ray (EDX) elemental mapping (In and Pt) of split crystals of **1a-1e**.

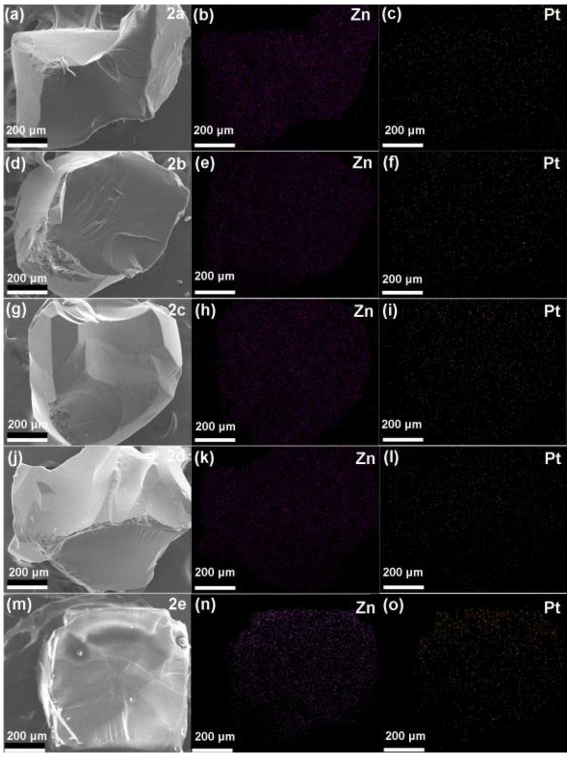

Fig. S5 Scanning electron microscope (SEM) image (a, d, g, j, m) and energy dispersive X-ray (EDX) elemental mapping (Zn and Pt) of split crystals of **2a**-**2e**.

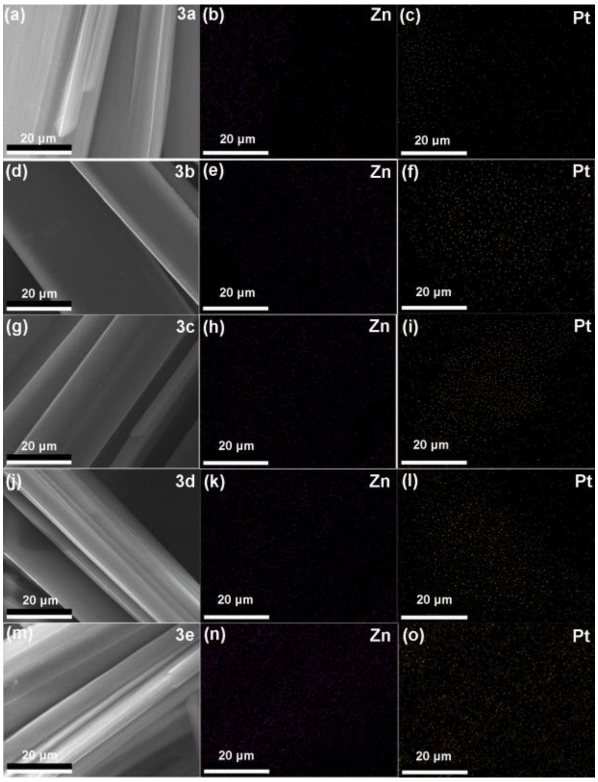

Fig. S6 Scanning electron microscope (SEM) image (a, d, g, j, m) and energy dispersive X-ray (EDX) elemental mapping (Zn and Pt) of split crystals of **3a**-**3e**.

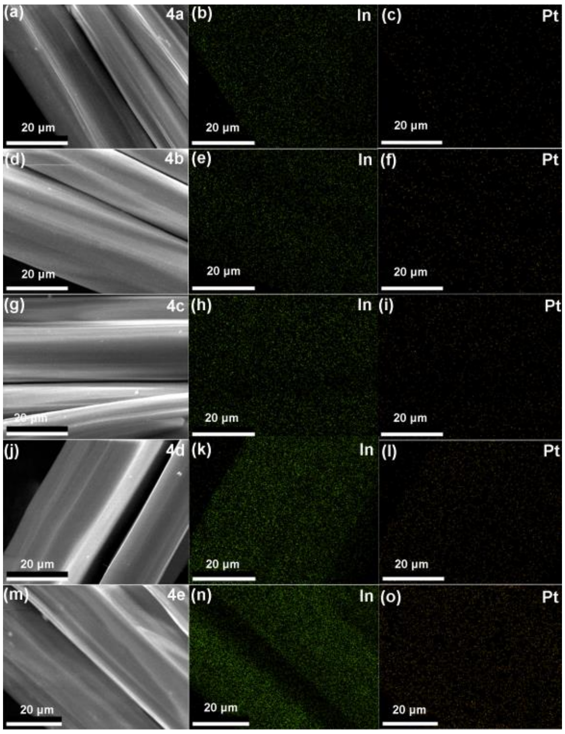

Fig. S7 Scanning electron microscope (SEM) image (a, d, g, j, m) and energy dispersive X-ray (EDX) elemental mapping (In and Pt) of split crystals of **4a**-**4e**.

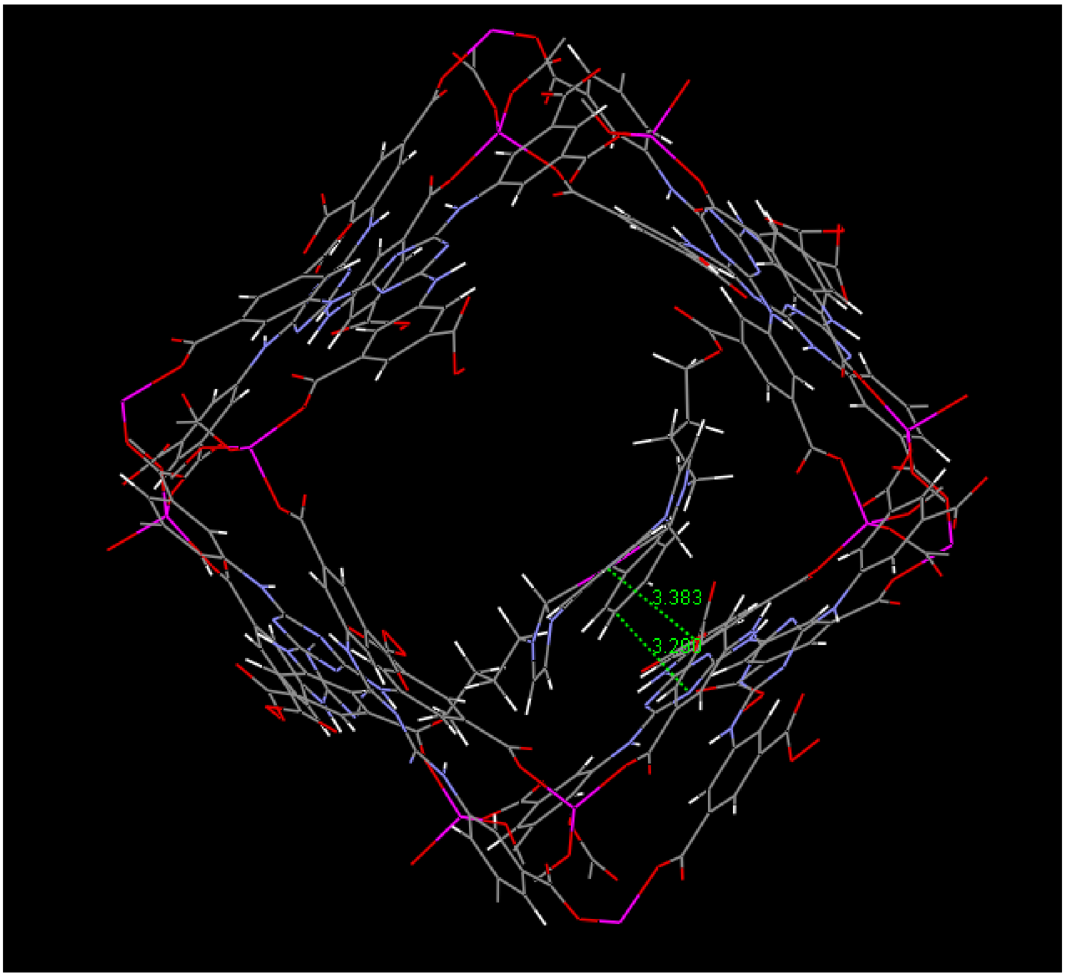

Fig. S8 The location site of **Pt1** in MOF2.

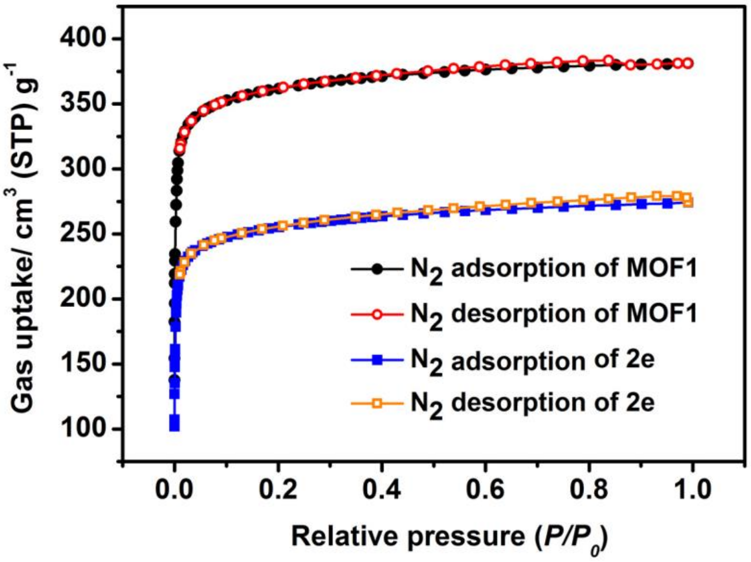

Fig. S9 Nitrogen sorption isotherms of MOF1 and **2e**.

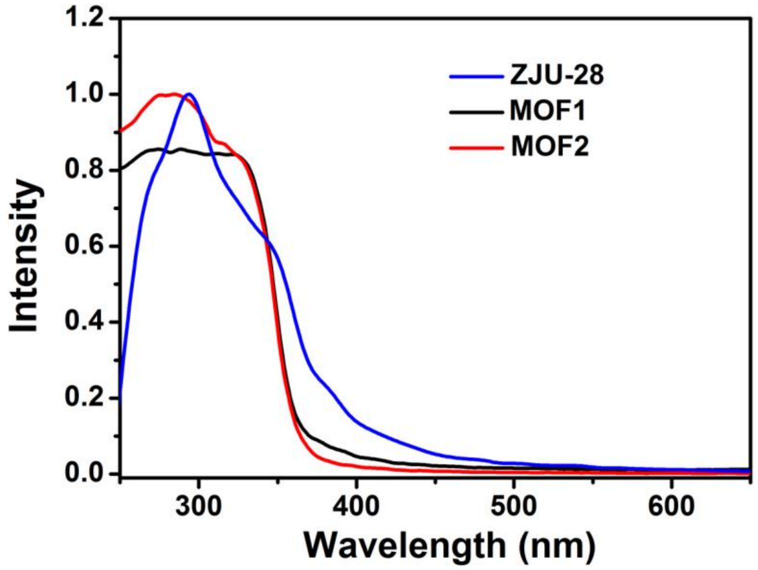

Fig. S10 Electronic absorption spectra of MOFs.

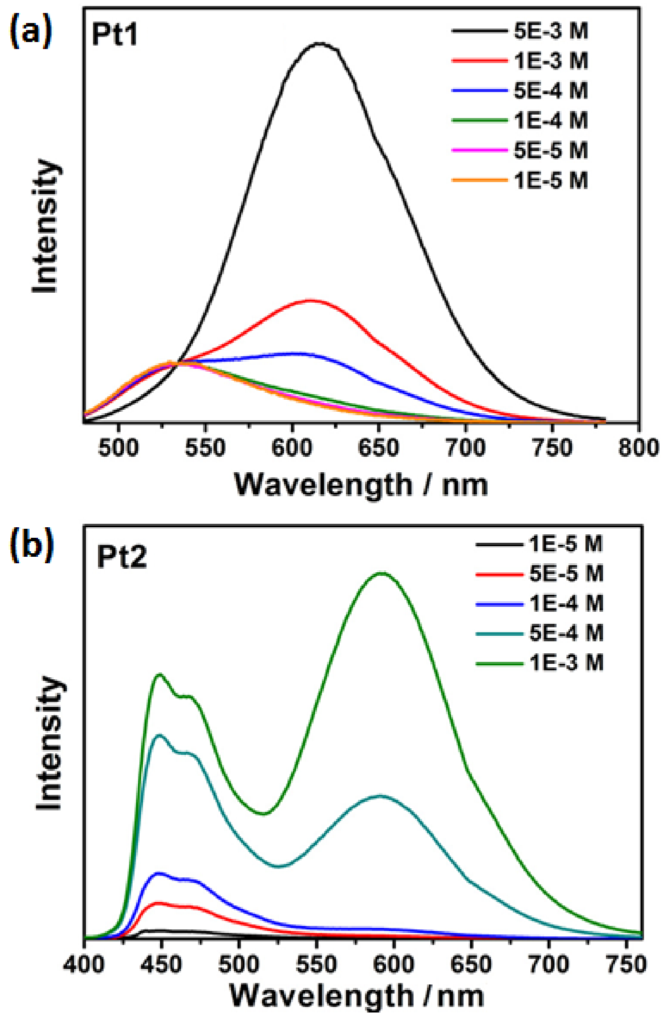

Fig. S11 Emission spectra of (a) **Pt1** and (b) **Pt2** in degassed MeCN upon excitation at 380 nm.

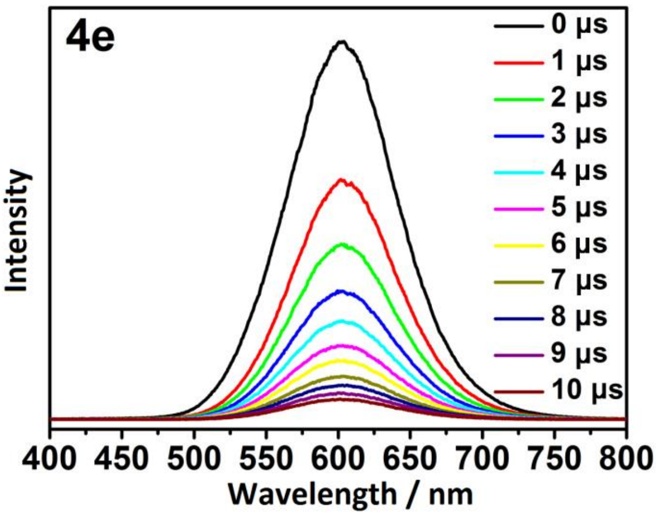

Fig. S12 Time-resolved emission spectra of **4e** in open air at room temperature.

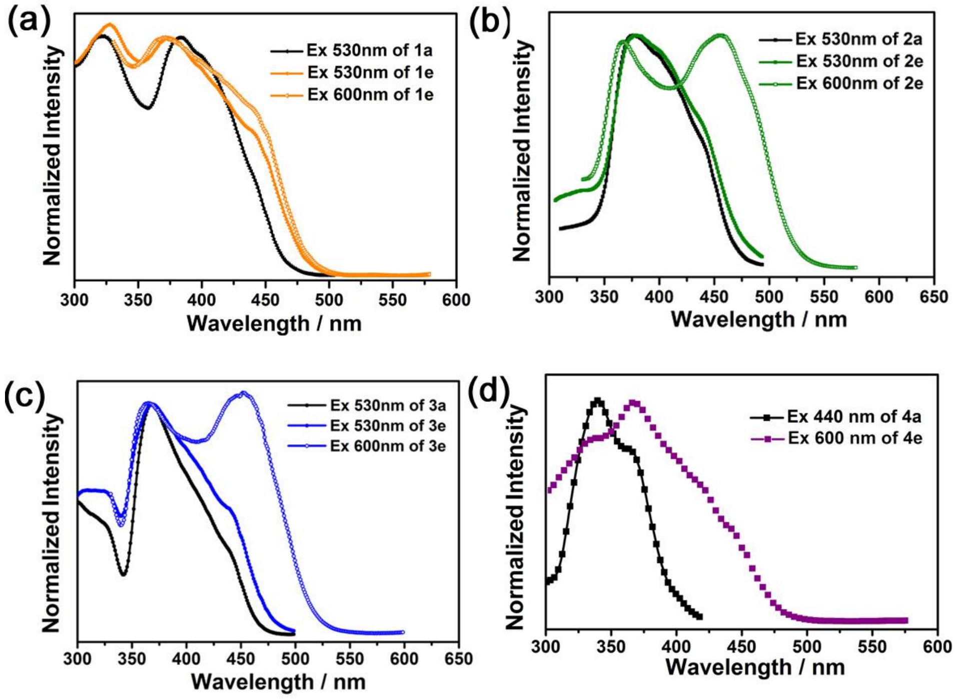

Fig. S13 Excitation spectra of Pt^{II}@MOFs at specified emission wavelengths in open air at room temperature.

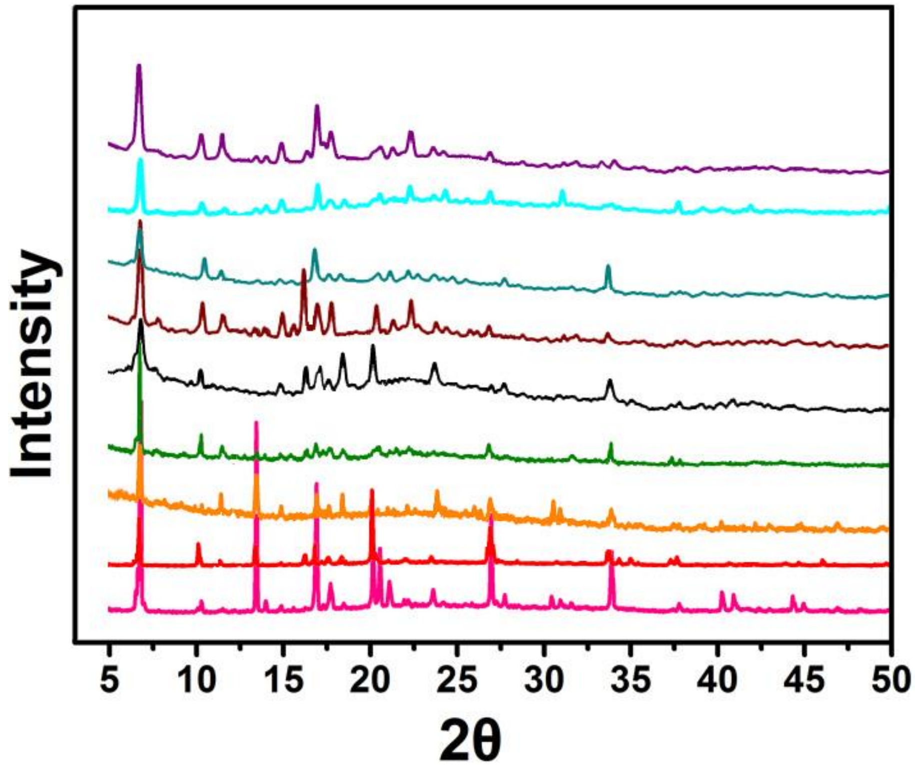

Fig. S14 PXRD patterns of as-synthesized ZJU-28 (pink), **1d** (red), and **1d** after catalysis for reaction III (orange), reaction IV (green), reaction V (black), reaction VI (wine), reaction VII (dark cyan), reaction VIII (cyan), reaction IX (violet).

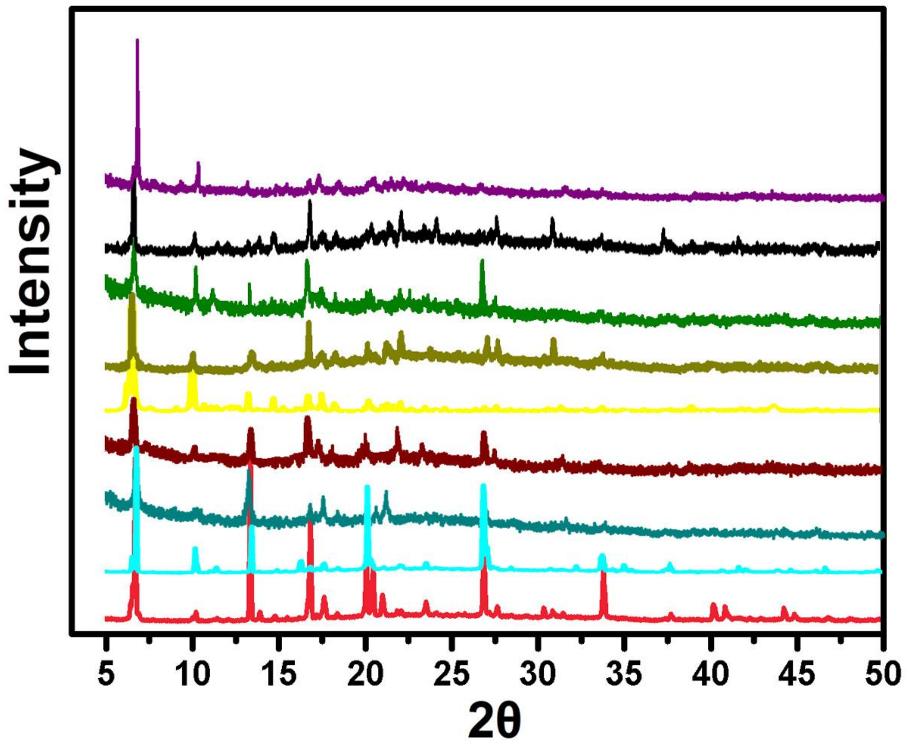

Fig. S15 PXRD patterns of as-synthesized ZJU-28 (red), **4d** (cyan), and **4d** after catalysis for reaction III (dark cyan), reaction IV (wine), reaction V (yellow), reaction VI (dark yellow), reaction VII (green), reaction VIII (black), reaction IX (violet).
